# CustomKinFragLib: Filtering the Kinase-Focused Fragmentation Library

**DOI:** 10.1021/acsomega.5c12231

**Published:** 2026-03-06

**Authors:** Paula Linh Kramer, Katharina Buchthal, Dominique Sydow, Katharina Sonja Leo, Andrea Volkamer

**Affiliations:** † Data Driven Drug Design, Center for Bioinformatics, 9379 Saarland University, Campus, 66123 Saarbrücken, Germany; ‡ In Silico Toxicology and Structural Bioinformatics, Institute of Physiology, Charité—Universitätsmedizin Berlin, Charitéplatz 1, 10117 Berlin, Germany

## Abstract

Protein kinases play a crucial role in key regulatory cell processes and are known to be dysregulated in diseases such as cancer and autoimmune disorders. Hence, protein kinases represent a vital drug target class. To meet the challenge of designing novel kinase inhibitors, fragment-based drug discovery (FBDD) has already shown great promise. The kinase-specific fragment library KinFragLib is a data-driven FBDD approach providing a powerful subpocket-specific framework for creating potentially feasible kinase inhibitors through subpocket-guided enumeration and combination of fragments. However, traversing the whole recombination space is computationally infeasible. Here, we introduce CustomKinFragLib, a curation-focused and user-oriented pipeline that builds on the existing KinFragLib framework. Building on the underlying fragmentation methodology, CustomKinFragLib contributes a systematic post hoc reduction and filtering strategy to generate a smaller, more tractable, and synthesis-friendly fragment set. The pipeline integrates literature-derived drug-relevant filters, including assessments of synthetic accessibility, matching to commercially available building blocks, and availability of retrosynthetic pathways. It also considers molecular properties often associated with drug-likeness and removes fragments containing unwanted substructures. Applying these curated filters reduces the original KinFragLib from 9131 to 837 fragments while retaining diverse fragments with drug-like properties and high synthetic tractability, and providing a practical fragment set suitable for downstream design workflows. Our pipeline is easily customizable, allowing for modifications or exclusion of filters based on the user’s preferences. The code and data set are available at https://github.com/volkamerlab/KinFragLib.

## Graphical abstract

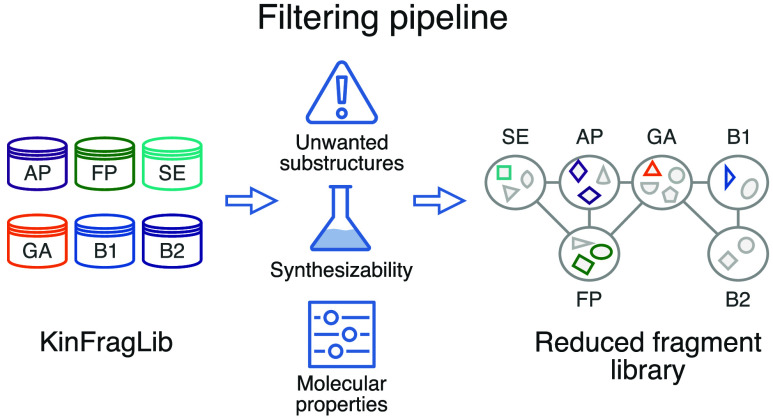

## Introduction

The human protein kinase family comprises 518 different kinases,^1^ representing one of the largest protein families. Kinases are involved in cell signaling processes since they bind adenosine triphosphate (ATP) and induce phosphorylation of proteins through the transfer of the terminal phosphate of ATP to a protein substrate. Consequently, kinases are often implicated in diseases such as cancer^2,3^ and the search for novel kinase inhibitors is of high pharmaceutical relevance. Kinases have been extensively studied, resulting in 6738 available protein–ligand complexes (recorded in the KLIFS database,^4^ June 2025), and 85 FDA-approved kinase inhibitors to date.^5^ However, only 22 kinase families^5^ have been targeted so far, showcasing the need for further inhibitors. Moreover, drug resistances pose a major challenge, highlighting the relevance of identifying novel kinase inhibitors.

Fragment-based drug design (FBDD) poses a promising way to approach the challenge of identifying novel drug candidates. The main mechanisms to generate novel compounds in FBDD consist of growing, merging, and linking of fragments. Fragment growing is the iterative addition of fragments to build a ligand. Fragment merging combines two or more promising fragments into one, preserving their favorable properties. Fragment linking connects such fragments using a linker that maintains their individual characteristics.^6^ FBDD has shown great promise in the search for novel kinase inhibitors, leading to the discovery of multiple FDA-approved inhibitors.^6,7^ Choosing meaningful fragments is crucial to this approach as they are the basis for all generated compounds. One way to generate meaningful fragments is to decompose relevant compounds into fragments and create a kinase-specific fragmentation library.^8,9^

In the Kinase-focused Fragmentation Library (KinFragLib),^10^ we extract fragments from kinase-ligand crystal structures assembled from the KLIFS database.^11^ Our goal is to group all fragments based on their position within the binding pocket. To achieve this, we dissect the ATP binding pocket into six functionally relevant subpockets, using the pocket information provided by KLIFS^4^ and from the literature.^10^ We assign fragments to subpockets based on geometric proximity. In addition, we also record the subpocket(s) to which the fragment was originally connected. The annotated fragments present a promising starting point for recombination to design novel kinase inhibitor candidates. However, the chemical space of fragment recombinations spans up to a billion possible compounds, which is computationally too expensive to traverse. One way to tackle this combinatorial space is to restrict the number of fragments to drastically reduce the number of possible recombinations while retaining relevant fragments. For this purpose, we present CustomKinFragLib, a tool to filter fragments based on undesirable substructures from the literature, molecular properties, and synthesizability. Using default parameters, CustomKinFragLib reduces the original KinFragLib^10^ set from 9131 to 837 fragments, spread over six different subpockets (see Figure 1). The filtering pipeline can be easily used and adapted, e.g., by changing the parameters or removing individual filters to fit different use cases. Each filter in our pipeline is optional and can be excluded, giving the user the option to create a custom filtering setup.

**Figure 1:**
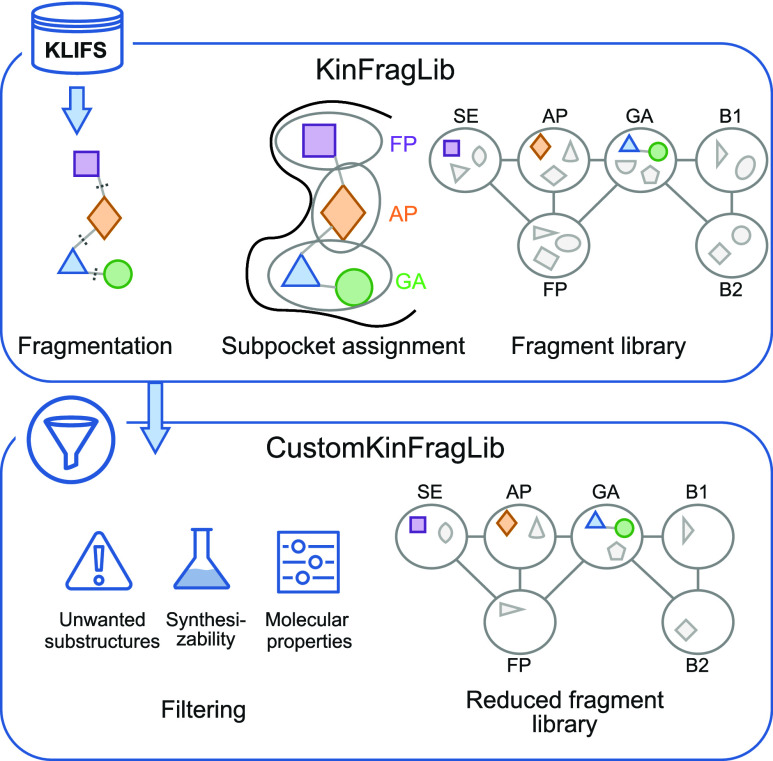
CustomKinFragLib overview. Kinase-ligand complexes from KLIFS are used to generate the KinFragLib fragment library. We fragment the ligands and assign them to the closest subpocket. To reduce the library, we apply different filters based on unwanted substructures, synthesizability, and molecular properties.

## Data and Methods

### Recap: KinFragLib Methodology and Available Fragments

KinFragLib^10^ is a fragmentation library incorporating structural kinase data from the Kinase–Ligand Interaction Fingerprints and Structures (KLIFS)^11^ database. The kinase-specific fragments originate from known kinase inhibitors and are annotated with their closest binding subpocket. The final fragmentation library contains 9131 fragments spanning six subpockets. In the following, we will give an overview of the KinFragLib methodology, with a focus on the subpocket definition and fragmentation procedure, as a basis for the introduction of CustomKinFragLib.

#### Kinase Binding Pocket Annotation

The binding pocket of protein kinases is structurally well conserved and is defined by 85 residues (see Figure 2). Kinases typically consist of two domains: the N-terminal lobe containing multiple β sheets and the C-terminal lobe mostly consisting of α helices.^12^ The catalytic cleft is located between these two lobes, which is the binding site for most kinase inhibitors. It consists of the front cleft, the gate area, and the back cleft. The front cleft can be divided into the adenine pocket (AP subpocket), which includes the ATP binding site, the front pocket (FP subpocket), and the glycine-rich loop. The gate area (GA subpocket) is situated between the αC-helix and the DFG-motif, the latter playing a significant role in identifying an active (DFG-in) or an inactive (DFG-out) conformational state of the kinase.^4^ The αC-helix is often anchored with a protein–protein salt bridge between residue 17 (in β sheet) and residue 24 (in the αC-helix). The salt bridge leads to a small shift of the whole helix, often observed in the active conformation of a kinase.^13^ The back cleft is positioned next to the αC-helix and contains a larger hydrophobic region (back pocket).

**Figure 2:**
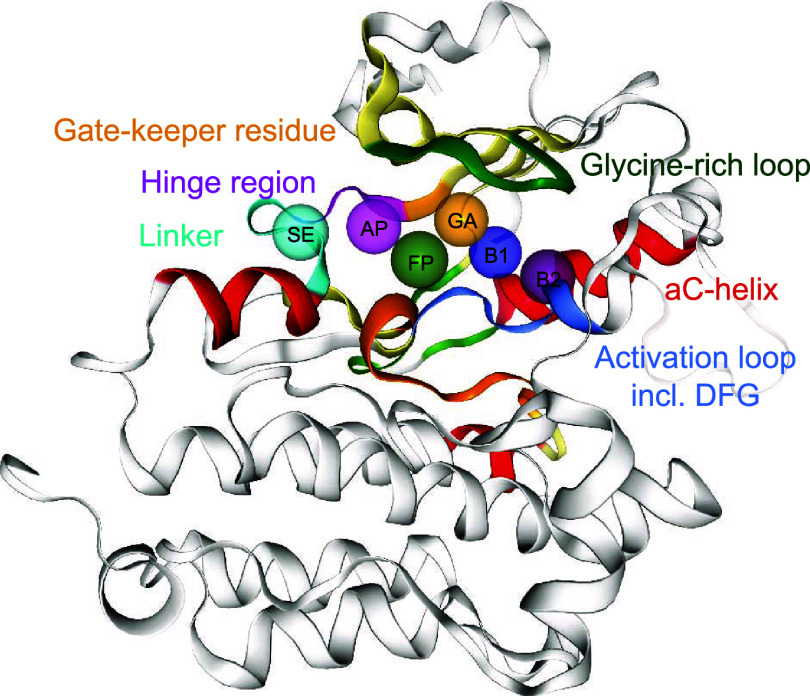
Exemplary EGFR kinase structure (PDB:4HJO) with key structural features and the six subpockets as defined by KinFragLib.

Based on these structural features, the binding pocket can be divided into six functionally distinct subpockets. The adenine pocket (AP) is located around the hinge region and is adjacent to the front pocket (FP) and the gate area (GA). Additionally, we have defined a solvent-exposed subpocket (SE pocket) adjacent to the AP and FP pocket. We divided the back cleft into two back pockets (B1 and B2 subpockets) in KinFragLib^10^ (see Figure 2).

#### Ligand Fragmentation

To create the fragmentation library, we consider all human kinase-ligand complexes from KLIFS,^4^ which are annotated to be in a DFG-in conformation (4609 PDB structures, December 2023). The bound ligands are dissected using the BRICS rules,^14^ which fragment compounds at retrosynthetically meaningful bonds defined by a set of predefined chemical rules. At each bond selected for fragmentation, the originally connected atom is replaced by a dummy atom, indicating the connection point at the respective fragments. Each fragment is annotated with a chemical environment. The fragmentation rules can be derived from a compatibility graph of these environments.^14^ These rules ensure a chemically meaningful fragmentation in which functional groups stay intact. It also increases the synthetic feasibility when recombining these fragments while maintaining the chemical environment compatibility. Hence, we annotate each connection point of the fragment with its chemical environment so that only BRICS-informed recombinations can be enforced. After fragmentation, each fragment is assigned to the closest subpocket in the kinase binding pocket according to the distance to the geometric subpocket centers. Since multiple neighboring fragments can be assigned to the same subpocket, these fragments are merged into a larger and more representative subpocket-specific fragment. All fragments that are located more than 8 Å away from any subpocket center are added to an additional subpocket pool X.

### Data-Driven Reduction of Fragment Space: CustomKinFragLib

With FBDD, we can generate novel drug candidates through fragment recombinations from KinFragLib. Since traversing the entire fragment recombination space of KinFragLib is computationally too expensive, we aim to reduce the fragment library by applying multiple filtering steps, leading to a reduced, drug-like fragmentation library. By default, all filters described in this section are applied for library reduction, but the user can also specify which filters, parameters, and thresholds should be applied, resulting in a more or less stringent collection of fragments. Our customizable filtering pipeline is available on GitHub at https://github.com/volkamerlab/KinFragLib.

#### Prefilters

The first filtering step comprises the removal of duplicated or unfragmented fragments as recommended in KinFragLib.^10^ We remove all duplicated fragments per subpocket based on exact matches of the standardized InChI (International Chemical Identifier) of the fragment (after removal of the dummy atom). Furthermore, we remove unfragmented structures, since they do not contain connection points and might cover multiple subpockets, making a useful subpocket assignment impossible. All fragments in subpocket X are removed, as well as fragments in other subpockets that only have connection points with subpocket X, since they cannot be recombined with any of the standard subpockets.

#### Unwanted Substructures

With these filtering steps, we remove molecules containing substructures with potentially undesirable properties. The two most widely used collections of unwanted substructures in the field of drug design are PAINS^15^ and substructures by Brenk et al.^16^ Pan Assay Interference Compounds (PAINS)^15^ provides a list of 480 substructures that have been observed to produce misleading results (‘false positives’) during high-throughput screening. Compounds containing these structures have been shown to interfere with the assay. Thus, the compounds appear to be active (‘hits’) but are not truly effective against the target of interest. Therefore, they are not suitable starting points for a drug development pipeline.

Brenk et al.^16^ have also collected a list of 105 substructures that potentially exhibit unwanted functionalities, such as mutagenic or highly reactive substructures, substructures with undesired pharmacokinetic properties, as well as PAINS. Both of these lists, which may contain some overlaps, have been included in our filtering steps, but depending on the users’ preferences, the filters can also be excluded. Fragments with matching substructures are removed from the fragment library.

#### Compound Properties

Similarly to the widely used Lipinski’s Rule of Five^17^ for bioavailability, a set of diverse, active fragments has been analyzed to derive common properties of fragments using RDKit functionalities.^18^ They have been defined as the Rule of Three^19^ and they state that the molecular weight (MW) should be less than 300 Da, the number of hydrogen bond acceptors (HBA) and donators (HBD) should be 3 or less, and the octanol–water partition coefficient (log *P*) should be less or equal to 3. In addition, the number of rotatable bonds (NROT) and the polar surface area (PSA) are also often considered drug-like criteria, with NROT ≤ 3 and PSA ≤ 60 Å^2^. By default, only the fragments meeting all criteria are kept in the fragmentation library. The number of allowed rule mismatches can be specified by the user.

As a second filtering step focusing on the compound’s physicochemical properties, the widely used Quantitative Estimate of Druglikeness (QED) filter^20^ is applied. It combines molecular properties describing drug likeliness into one continuous score between 0 and 1. The molecular properties covered are molecular weight, log *P*, hydrogen bond acceptors, hydrogen bond donors, polar surface area, rotatable bonds, number of aromatic rings, and structural alerts. Each property is modeled by an asymmetric double sigmoidal function with parameters defined based on ≈770 orally administered approved drugs. The final QED score is derived from a weighted geometric mean of all these functions. The weight of each function should be proportional to the significance of this property toward the drug likeness. The best combination of weights for the functions is determined using the maximum information content calculated by the Shannon entropy. While the QED score was originally calculated for full ligands, we analyzed the usability for fragments. We extracted fragments from FDA-approved kinase inhibitors and inhibitors in clinical trials by querying the PKIDB^21^ database (accessed September 2025), since we assume these to be drug-like. We calculated the QED scores for all fragments that are also included in KinFragLib and chose the lower 25% quantile of QED scores (0.464) as default threshold.

#### Synthetic Accessibility

As further filtering criteria, synthesizability is considered. For drug development, promising molecules should also be synthesizable in an easy, fast, and cost-efficient manner, e.g., to accelerate drug development. Hence, starting with synthetically accessible fragments can improve the synthesizability of the enumerated molecules. We present three different filters to increase the synthesizability in our fragment library: the SYBA score,^22^ classifying compounds according to the synthetic accessibility, a substructure search of commercially available buyable fragments, and ASKCOS^23^ for analyzing the retrosynthesizability of fragment pairs.

##### SYBA

There are multiple scores trained to predict the synthesizability of molecules, such as the SAScore,^24^ the RAscore,^25^ and the SYnthetic Bayesian Accessibility^22^ (SYBA) score. While we have selected SYBA, the modularity of our pipeline makes the integration of other scores straightforward. SYBA is a fragment-based synthesizability predictor^22^ that is trained based on the frequency of a fragment being present in easy and hard-to-synthesize compounds in the ZINC15 data set. The SYBA score of a fragment is based on the logarithmic ratio of the probability that it is hard to synthesize and the probability that it is easy to synthesize. A score of 0 means that both probabilities are equal, a more positive score indicates an easier-to-synthesize molecule, and a more negative score indicates that the molecule is harder to synthesize. We remove fragments with a negative SYBA score from the library.

##### Enamine

The second synthesizability filter aims to remove fragments that might not be commercially available. Hence, we compare the KinFragLib fragments—stemming from cutting known cocrystallized ligands—with commercially available building blocks by Enamine. The Enamine REAL space currently (queried on January 2024) holds more than 48 billion make-on-demand molecules, which are stated to be synthesized within 3–4 weeks with a success rate of more than 80%.^26^ The Enamine REAL Space is based on roughly 1.3 million Enamine Building Blocks (as of January 2024), which are available for download on the Enamine Web site. We have implemented a substructure search on the Enamine Building Block database to compare the kinase fragments with the Enamine space. For each KinFragLib fragment, we checked whether it is a substructure of any Enamine building blocks with the rdkit.Chem.rdSubstructLibrary function in the RDKit library.^18^ For the substructure search, we remove all connection points by replacing the dummy atoms with hydrogens. We exclude fragments that lack substructure matches with Enamine building blocks, as their absence in this extensive commercial library suggests that they may be more challenging to synthesize. Additionally, compounds recombined from this fragment library are more likely to be in the Enamine REAL space or to have a high similarity to commercially available compounds.

##### ASKCOS

This filter assesses the retrosynthesizability of fragment pairs using ASKCOS,^23,27^ an open-source computer-aided retrosynthesis analysis tool. ASKCOS has first collected ≈150k frequent single step reaction templates from the Reaxys database. A feedforward neural network is trained to predict the most suitable transformation from the database for a given molecule. Additionally, a neural network classifier trained on positive and negative reaction examples indicates the prediction quality. First, we generate all potential molecules that can be formed from two matching KinFragLib fragments. Then, we utilize ASKCOS to determine if these molecules can be synthesized via a one-step reaction. To do this, we create fragment pairs from our fragment library, considering a pair as valid if it fulfills the following criteria:

- The fragments’ attachment points need to point to adjacent subpockets. For example, fragment 1 originates from subpocket AP, and fragment 2 is from subpocket FP. The attachment point of fragment 1 points to the FP subpocket, and the attachment point of fragment 2 points to the AP subpocket. The fragments’ subpockets, attachment points, as well as neighboring subpockets, are annotated in KinFragLib (as depicted in Figure 3A).
- The BRICS^14^ environment types of the two fragments need to be compatible, indicating a retrosynthetically meaningful bond (see an example in Figure 3B).
- Both fragments need to fulfill all previous filtering criteria (no unwanted substructures, drug-like properties, and synthesizability). This is done to reduce the number of fragment pairs, to stay within a reasonable computational runtime when querying ASKCOS.

**Figure 3:**
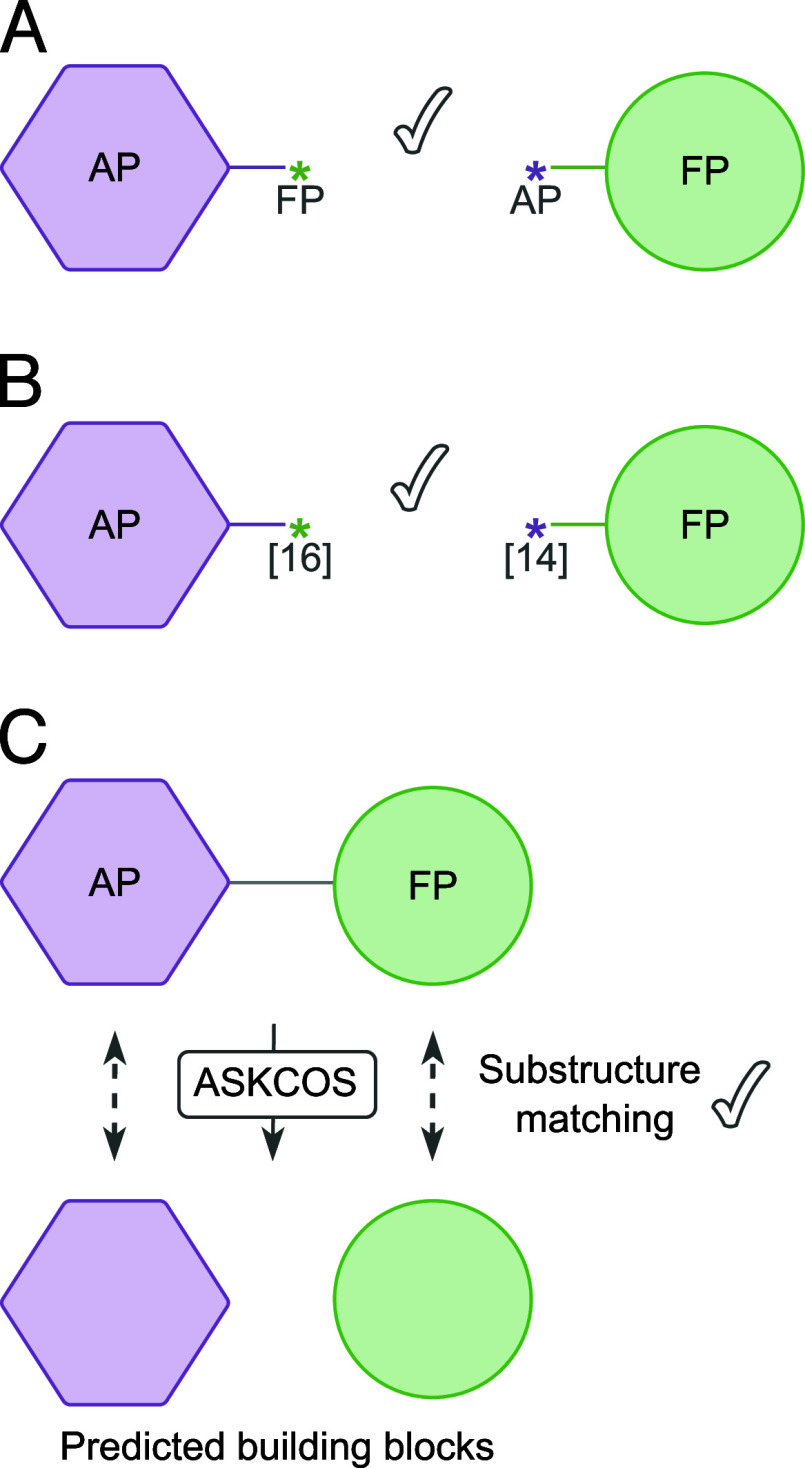
Pipeline for performing the ASKCOS filtering. (A) Checking for subpocket compatibility. (B) Checking for BRICS environment compatibility. (C) Fragment recombination, followed by ASKCOS predictions. A substructure matching is performed between the fragments suggested by ASKCOS and our fragments.

After identifying all valid fragment pairs, we evaluate whether a one-step retrosynthetic route can be found to synthesize the compounds formed by these pairs. If a retrosynthetic path is found, we compare the precursor fragments suggested by ASKCOS to our fragment pair. If our fragments are substructures of the precursors, we classify the fragment pair as retrosynthetically feasible (see Figure 3C). We retain only those fragments in our library that contribute to at least one retrosynthetic route. All parameters used for the ASKCOS query are listed in Supporting Table S1.

### Diversity Evaluation of Fragment Library

To assess the diversity of our fragment library, we perform two analyses: one based on Tanimoto distances and another using Shannon entropy.

#### Tanimoto Distance

We calculate the average pairwise Tanimoto distances using RDKit^18^ of all fragments per subpocket. The Tanimoto distance is defined as 1–*tanimoto similarity* and is calculated using the RDKit fingerprint. The average and standard deviation of the Tanimoto distance is reported.

#### Shannon Entropy

A second diversity analysis is performed based on the Murcko scaffolds,^28^ where all fragments are reduced to the core ring structure without any side chains. For this analysis, not all fragments are considered since not all fragments contain a ring structure. We measure the information-based entropy of these scaffolds using the Shannon entropy^29^

$$\mathrm{SE}=\sum_{i=1}^{n}p_{i}\mathrm{lo}g_{2}p_{i}$$

for the *n* most frequent scaffolds. *p*_*i*_ is defined to be

$$p_{i}=\frac{c_{i}}{P}$$

where *P* denotes the total number of compounds and *c*_*i*_ denotes the number of compounds with scaffold *i*. The standardized Shannon entropy (SSE) is defined as

$$\mathrm{SSE}=\frac{SE}{log_{2}n}$$

which scales the entropy to a range between 0 and 1, with 1 indicating a high entropy among the scaffolds.^30^

### Molecule Enumeration Sets

To assess molecule-level consequences of fragment-level filtering, we generated two recombination sets and calculated molecular properties for each. The first set consists of molecules recombined from CustomKinFragLib fragments. For comparison, a second set was created from fragments rejected during the filtering pipeline. To reduce computational runtime, we randomly sampled 80 fragments from each subpocket. Note that some subpockets contain less than 80 fragments (see Supporting Table S2 for fragment numbers). The AP subpocket was defined as the core subpocket, ensuring that each recombined molecule contains an AP fragment. Additionally, we restricted molecules to contain a maximum of four fragments, to keep the molecular size in a reasonable range. We then enumerated all valid recombination following the subpocket connection rules and the BRICS^14^ rules, resulting in ≈770,000 ligands in the rejected-fragment set and ≈780,000 ligands in the CustomKinFragLib set. Because combinations of four fragments dominate the enumeration space, this distribution may introduce bias. To mitigate this and to better reflect the fragment-count distribution observed in KLIFS ligands (see analysis in KinFragLib^10^), we sampled molecules containing two to four fragments in proportions matching the KLIFS data set.^4^ This results in recombination sets of ≈20,000 ligands each.

To evaluate whether filtering a fragment library improves molecular properties of recombined molecules, we inspected the recombination sets based on drug-likeness and synthesizability. For the drug-likeness, we applied the well-known Lipinski’s Rule of Five,^17^ the Veber’s rule and the QED^20^ score. To assess the synthesizability, we calculated the SYBA^22^ score and compared the molecules to the Enamine REAL Space. Since we compared the fragments to the Enamine building blocks, we expect a certain similarity between the recombined molecules and the Enamine REAL Space, which is built using the Enamine building blocks. We used SpaceLight^31^ to extract the most similar molecule from the Enamine REAL Space for each query molecule. We did not analyze the unwanted substructures on a molecule-level, since this is an injective property, meaning that if an unwanted substructure is present in a fragment, it will also be present in the full molecule containing this fragment. Furthermore, we skipped ASKCOS for this analysis, since this is already a molecule-level filter, where pairs of fragments are recombined and queried.

## Results and Discussion of Fragment Library Reduction

In the following, we will first discuss the updated KinFragLib library and CustomKinFragLib library sizes. Then, we analyze the chemical space and the diversity of the reduced fragment library compared to the full library. Note that we discuss the findings for the pipeline here using default parameters, but the user can easily adapt the parameters to their needs.

### KinFragLib Update

We present an updated fragmentation library including all recently published structures (4609 PDBs) in KLIFS^11^ that were released until December 2023. Our fragmentation library is extended to 9131 fragments instead of roughly 7000 fragments, originating from 3232 kinase-ligand complexes compared to 2553 structures in the original publication^10^ (see Figure 4).

**Figure 4:**
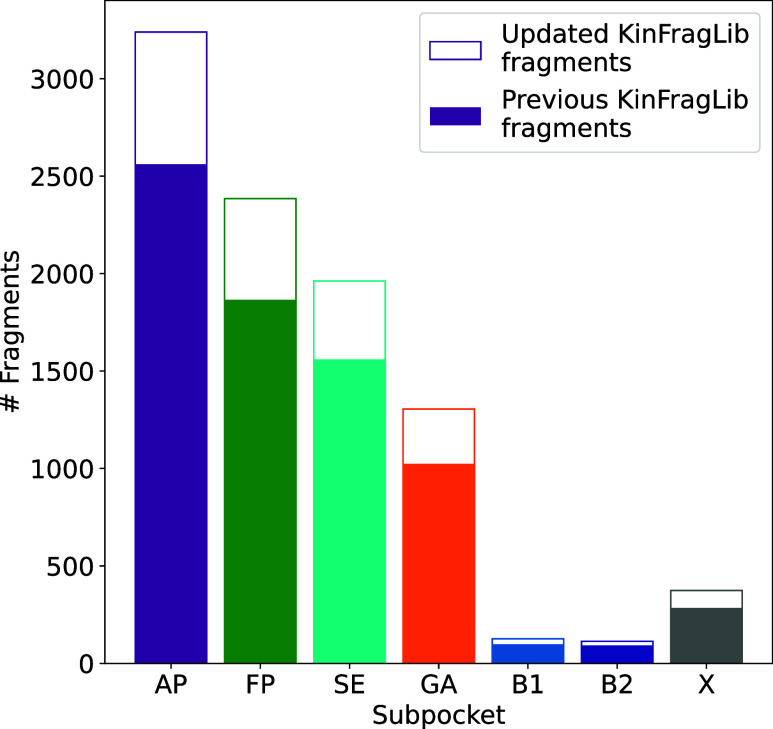
Fragment library size per subpocket of the previously published KinFragLib library compared to the fragment library containing an updated KLIFS version.

### CustomKinFragLib Size

To address the challenge of the combinatorial explosion when enumerating fragment libraries, we aimed to reduce the number of fragments while maintaining the most promising ones. Starting with 9505 fragments in KinFragLib, we reduced the space in two steps: First, we refined the fragment library by eliminating duplicate fragments, unfragmented ligands, and fragments that did not match any of the six predefined subpockets (pool X). This reduction leads to a fragment library size of 3414 fragments. Second, we further reduced the KinFragLib fragment space to 837 fragments with six custom-made filters detailed below (see Figure 5).

**Figure 5:**
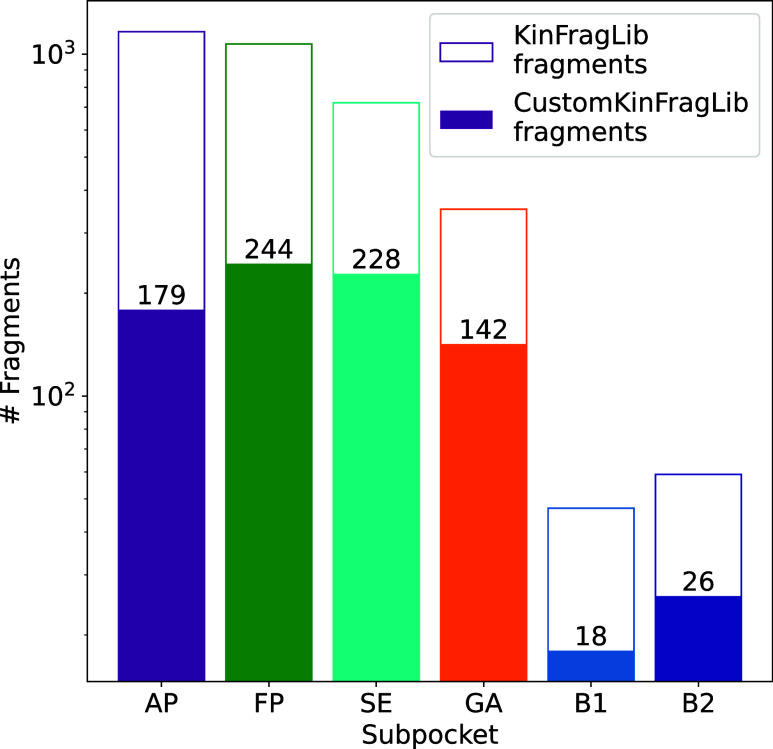
Comparison of number of prefiltered KinFragLib and CustomKinFragLib fragments per subpocket. The number of fragments is displayed on a logarithmic scale. The numbers on each bar represent the absolute number of CustomKinFragLib fragments per subpocket.

The CustomKinFragLib filtering cascade removes fragments from the library that contain unwanted substructures, are not drug-like, or are too complex to synthesize. Figure 10 shows the number of fragments removed by each filtering rule separately. We observe that four of the filtering steps retain the majority of fragments for all subpockets, whereas the other filters remove the majority of fragments. In the following, we exemplarily discuss the observations for the hinge binding pocket (AP) containing 1164 fragments before filtering. By applying the seven different filters, we reduce the number of AP fragments to 179. For instance, the PAINS filter for unwanted substructures (480 substructures are collected here) removes only 13 fragments (≈1.1%) in the AP subpocket. The Brenk filters (covering 105 substructures) remove 253 fragments in the AP subpocket. Figure 6 shows four different examples in which the unwanted substructure is highlighted.

**Figure 6:**
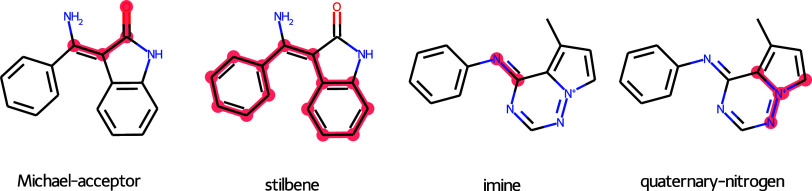
Examples of molecules containing unwanted substructures highlighted in red from Brenk et al.

Regarding the synthesizability filters, the SYBA score removes 131 fragments (11.3%) from the AP subpocket, indicating that the majority of fragments are predicted to be easier to synthesize. The distribution of the scores and the default cutoff are given in (Supporting Figure S1). Exemplary five molecules with a low SYBA score—indicating poor synthetic accessibility—are depicted in Figure 7. Three structures contain a macrocyclic substructure, implying a more challenging synthesis. Further molecules contain a chiral center (third molecule) or an exocyclic double bond (last molecule), both indicating a more challenging synthesis.

**Figure 7:**
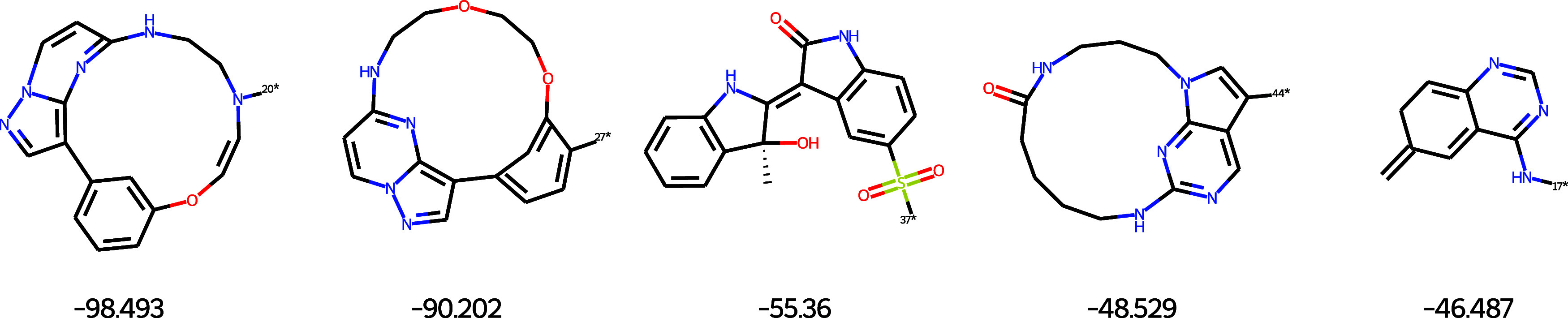
Examples of molecules predicted to be very hard to synthesize by SYBA.

In contrast, the Enamine building block filter removed around 667 fragments (57.3%) from the AP subpocket. This substantial reduction results from the stringent requirement that each KinFragLib fragment must have an exact substructure match within at least one of the 1.3 million Enamine building blocks. The fraction of fragments removed exhibited similar behavior in the different subpockets. Two fragments rejected by this filter are shown in Table 1 together with their most similar Enamine building block. In both cases, the nearest commercially available building block differs substantially, with a Tanimoto similarity below 0.3 using a topological, fragment-based connected substructure fingerprint (fCSFP).^31^ The lack of close analogs in Enamine suggests that these fragments may be hard to obtain commercially and potentially more difficult to synthesize.

**Table 1:**
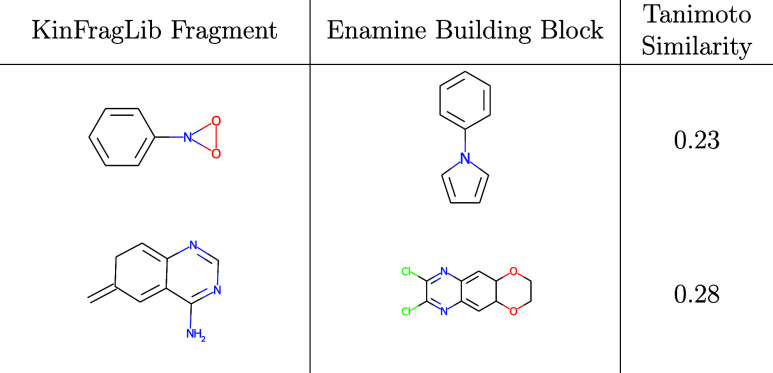
Examples of Fragments Rejected by the Building Blocks Filter^a^

a The most similar building block to the KinFragLib fragment has been added.

The Ro3 filter—aiming to filter out less drug-like fragments—removes around half (≈600) of the fragments from the AP library, since we have employed, by default, a strict Ro3 filtering by not allowing any rule mismatches. For each filter, we have added an example of a rejected molecule in Figure 8. Note that these example molecules also fail to fulfill other Ro3 filtering criteria—the full table is listed in Supporting Table S3. Furthermore, Figure 9 shows three exemplary molecules filtered out by the QED filter. All molecules were also flagged by the Brenk filter for unwanted substructures, in addition to some Ro3 filters not being fulfilled (for the first and third molecule) (Figure 9).

**Figure 8:**
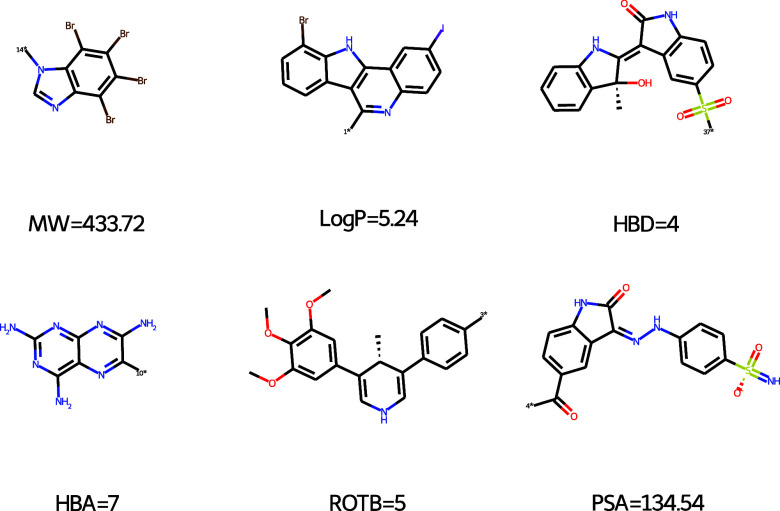
Examples of rejected molecules for each of the Rule-of-three filter.

**Figure 9:**
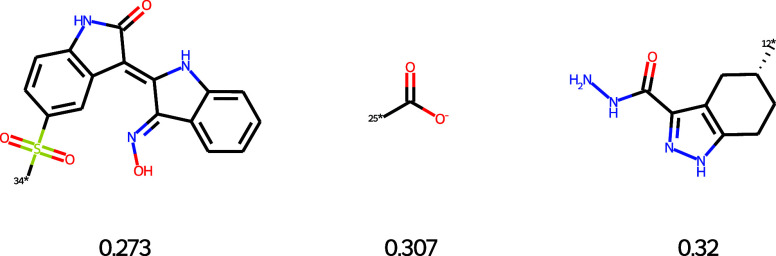
Examples of molecules with low QED score.

**Figure 10:**
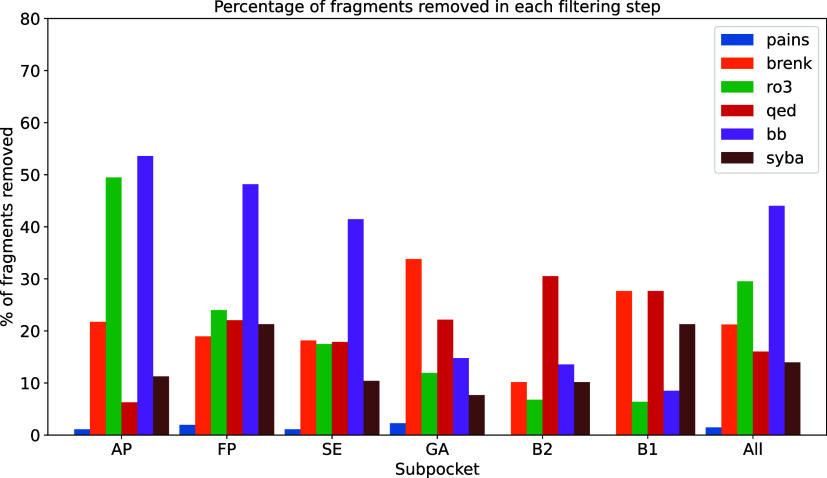
Percentage of fragments removed per subpocket and in total by each filtering step: Unwanted substructures (patterns from PAINS and Brenk et al.), drug-likeness (Rule-of-three (Ro3) and QED score), and synthesizability (Enamine building blocks (bb) and SYBA score).

The total number of fragments remaining after these filtering steps—when applied one after the other—for each subpocket is listed in Figure 11. Applying the retrosynthesizability filter ASKCOS^23^ is time-consuming, since we need to enumerate all fragment pairs beforehand, whereas all other filters are run on the fragments only. Therefore, we apply ASKCOS only to all fragment pairs passing the filtering rules shown in the previous (Figure 10). Using the web API of ASKCOS^32^ (accessed October 2025), we query 63,494 unique fragment pairs to find one-step retrosynthetic routes for each pair. An example of an ASKCOS query is shown in Figure 12, where our query molecule consists of two KinFragLib fragments. Among the five different predicted synthetic routes, at least one route has overlapping starting structures with the KinFragLib fragments, indicating the presence of synthetically feasible fragments. Applying this filter to our fragment library reduces the library further, but for most pockets, the majority of fragments are retained since they are part of a feasible retrosynthetic pathway (see Figure 11).

**Figure 11:**
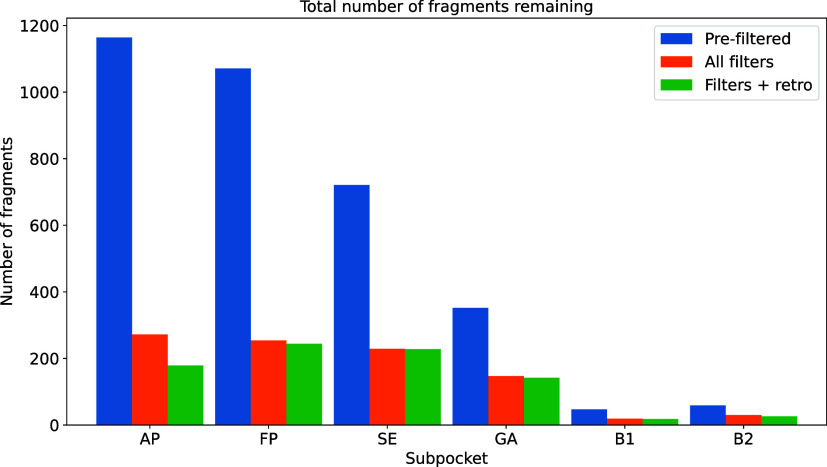
Number of fragments remaining after applying all filtering steps in Figure 10. *Filters + retro* contains the final number of fragments after applying the retrosynthesizability filter. This is only applied to the filtered number of fragments, due to the computational runtime.

**Figure 12:**
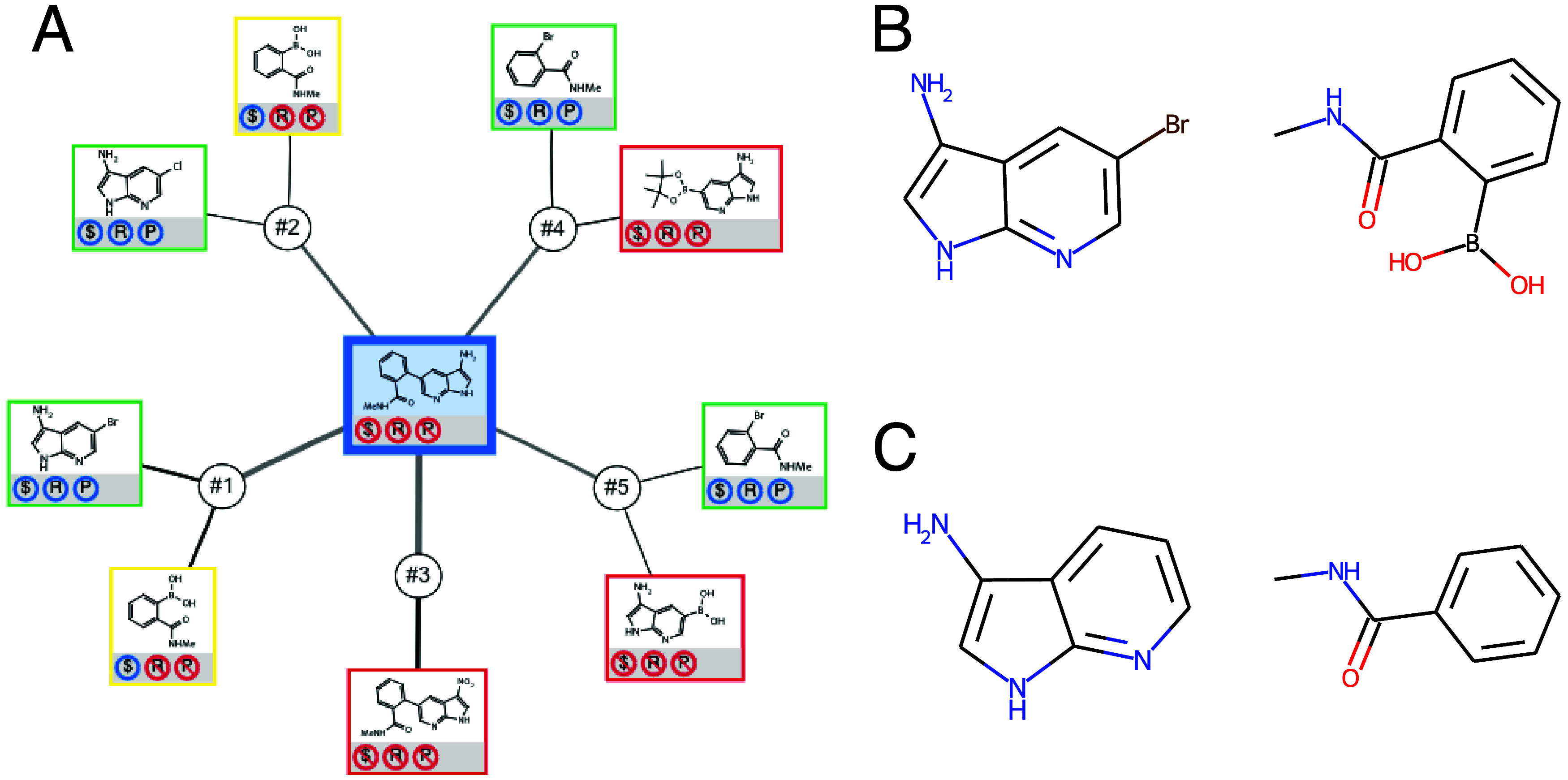
(A) ASKCOS query for one pair of fragments from KinFragLib resulting in multiple suggestions. ASKCOS collects information about the query and all precursor fragments, indicating whether it is commercially available, whether it has a history as a reactant (R), and as a product (P). Suggestion #1 contains the precursor fragments in (B) which overlap with our KinFragLib fragments for the query in (C). (A) represents a screenshot from the ASKCOS Web server^32^ with the results to our query (MIT license).

While the filters aim to reduce different unwanted properties of the fragments, we investigated whether the filters are redundant. Table 2 shows the number of fragments rejected by two different filters each. The most significant overlap between two filters is between the PAINS and the Brenk filters, which both check for unwanted substructures, and the Brenk filters also include substructures aiming to reduce PAINS. In addition, most of the molecules filtered out by the Ro3 filter lack substructure matches with the Enamine building blocks, suggesting that they are not easily synthesizable using the available commercial fragments. When applying the Ro3 filters directly to the Enamine building blocks, only roughly 40% passed, highlighting the strictness of the filters. This suggests a significant overlap between drug-like filters (as defined by Ro3) and commercial availability of fragments, which is expected, as commercial libraries often reflect drug-like design principles. For all other combinations, the majority of molecules filtered out seem to be unique to the respective filter, showing the importance of filtering for different aspects in our pipeline.

**Table 2:** Number of Fragments Jointly Filtered out by Two Different Filtering Criteria (Off-Diagonal) as well as by the Individual Filters (Diagonal)

	PAINS	Brenk	Ro3	QED	BB	SYBA
PAINS	50	44	30	12	25	3
Brenk		725	265	177	295	176
Ro3			1008	99	693	122
QED				547	177	177
BB					1649	276
SYBA						477

### Chemical Space Analysis

Since we drastically reduced the fragment library from 9131 to 837, it is crucial to retain a high diversity, guaranteeing that we still cover a wide chemical space. To visualize the chemical space, we use t-SNE, an approach transforming high-dimensional data into a two-dimensional representation.^33^ The comparison between prefiltered KinFragLib and CustomKinFragLib fragments reveals that most fragments still have a nearby fragment in the t-SNE plot included in CustomKinFragLib, indicating that a high diversity among the filtered fragments remains (Figure 13A).

**Figure 13:**
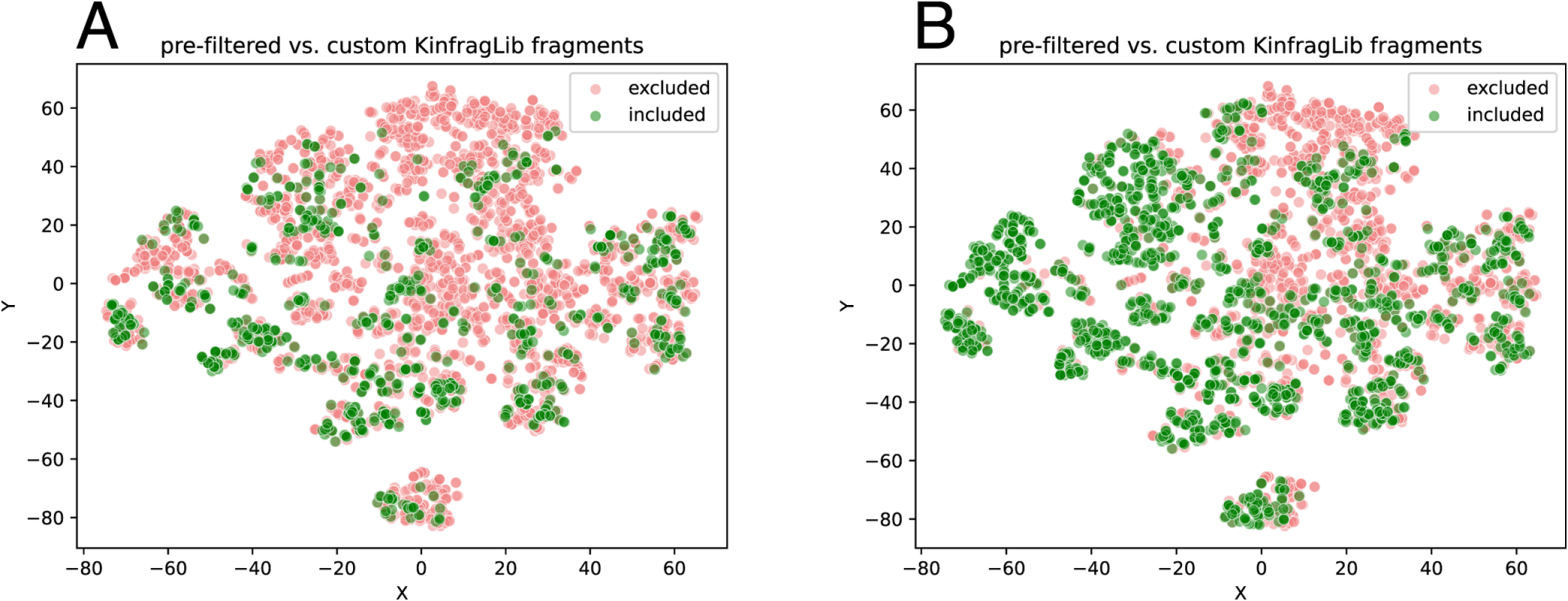
(A) t-SNE plot of prefiltered KinFragLib fragments and the overlap to CustomKinFragLib. Red indicates that the fragment is not included in CustomKinFragLib, while green represents fragments in CustomKinFragLib. (B) t-SNE plot of prefiltered KinFragLib fragments and the overlap to all fragments matching Enamine building blocks. Red indicates that the fragment is not included in the Enamine building blocks, while green represents fragments in Enamine.

However, we also observed that, particularly in the upper area of the t-SNE plot, more fragments are filtered out, resulting in lower coverage of that part of the chemical space. Consequently, we investigated which filtering criteria were responsible for removing fragments in that area. We found that the Enamine filter removes the majority of fragments in the upper part. Figure 13B illustrates the chemical space of the prefiltered KinFragLib library, where red points represent fragments filtered out due to not matching any Enamine building blocks. After visual inspection of the fragments, this part of the chemical space contains charged fragments, which are filtered because the Enamine fragments are mostly uncharged. Analogous figures for all other filters are provided in Supporting Figure S4. For each filter, we can observe that small clusters are filtered out, and each filter removes mostly different clusters in the t-SNE space.

#### Assessment of Fragment Diversity by Tanimoto Distance

As a measure of diversity, we also calculated the average pairwise Tanimoto distance between all fragments in the respective fragment library (prefiltered KinFragLib and CustomKinFragLib) using the RDKitFingerprint.^18^ The diversity of all fragments in the prefiltered KinFragLib library is 0.91 ± 0.09, while CustomKinFragLib has a diversity of 0.88 ± 0.11. Table 3 also contains the diversities for each subpocket in KinFragLib and CustomKinFragLib, respectively. Even though the fragment library was reduced to 25% compared to the prefiltered KinFragLib size, we observed only a slight loss of 0.03 in diversity.

**Table 3:** Two Diversity Measurements: Average Pairwise Tanimoto Distance Including Standard Deviation Per Subpocket and all Subpockets Merged for Pre-Filtered KinFragLib and CustomKinFragLib, Respectively^a^

	Tanimoto		SSE	
Subpocket	KinFragLib	Custom	KinFragLib	Custom
AP	0.86 ± 0.09	0.84 ± 0.12	0.88	0.91
FP	0.91 ± 0.09	0.90 ± 0.10	0.81	0.74
SE	0.90 ± 0.10	0.88 ± 0.12	0.81	0.73
GA	0.90 ± 0.11	0.85 ± 0.12	0.65	0.67
B1	0.91 ± 0.13	0.84 ± 0.15	0.68	0.84
B2	0.91 ± 0.11	0.88 ± 0.11	0.84	0.89
Total	0.91 ± 0.09	0.88 ± 0.11	0.78	0.70

a Average pairwise Tanimoto distance including standard deviation per subpocket and all subpockets merged for pre-filtered KinFragLib and CustomKinFragLib, respectively. Standardized Shannon entropy (SSE) of Murcko scaffolds per subpocket and for the whole fragment libraries (pre-filtered KinFragLib and CustomKinFragLib).

#### Diversity Assessment by Shannon Entropy

As a second diversity analysis, we calculated the Shannon entropy of the Murcko scaffolds of the fragments.^28^ Since all fragments without rings do not have a Murcko scaffold, these were excluded from this diversity analysis. In prefiltered KinFragLib, 8.7% of all fragments do not contain a ring and were excluded from this analysis. In CustomKinFragLib, only 3.7% of the fragments were excluded due to missing rings. Since the fraction of excluded structures is low for both fragment libraries, the Shannon entropy measure still represents a meaningful way of calculating diversity.

We observe high entropy among the fragments for each subpocket (Table 3), indicating a high level of diversity in KinFragLib as well as CustomKinFragLib. The high diversity observed based on fingerprints and scaffolds indicates that our filtering approach is an effective method for reducing a fragment library without losing essential parts of the space.

#### Kinase Coverage

To evaluate how well our fragmentation library represents the kinase inhibitor space, we analyzed the coverage of ligands across different kinases. This coverage analysis is performed separately for each subpocket to account for their distinct fragment distributions and characteristics. For each subpocket, we assume a kinase to be covered, if at least one ligand binding to this kinase contains a fragment included the respective fragmentation library. Figure 14 contains the kinase coverage for the AP subpocket. The figures for the other subpockets can be found in the Supporting Figure S5.

**Figure 14:**
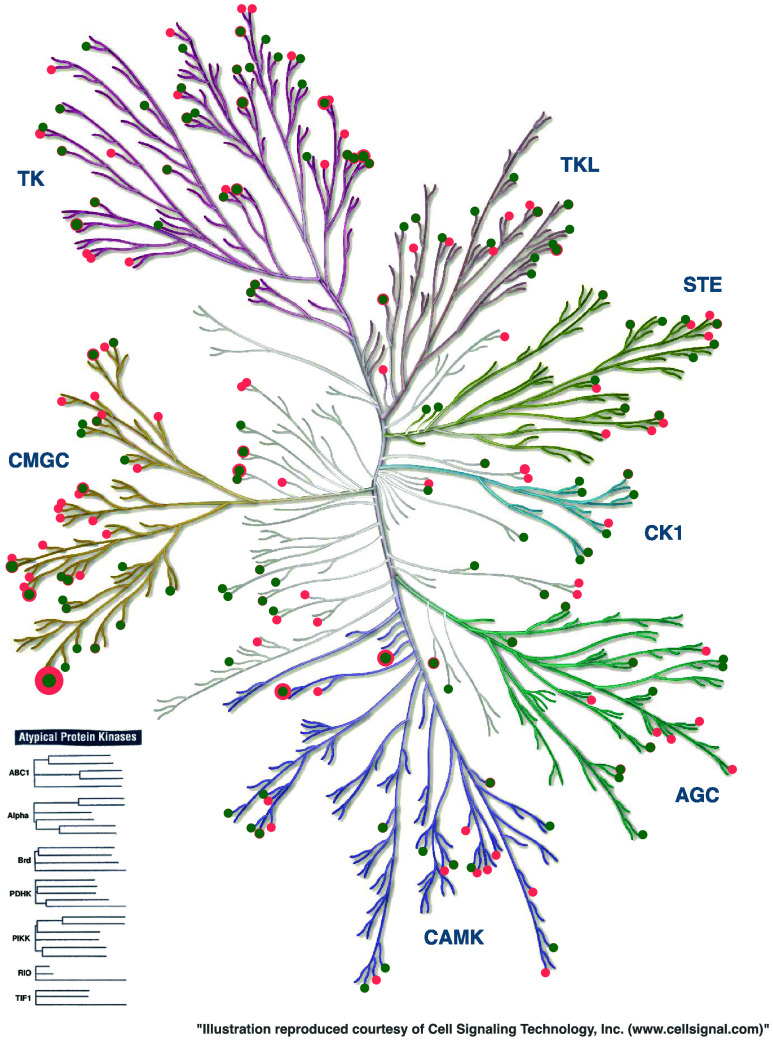
Kinome tree generated using KinMap.^34^ Ligands binding to the kinases that contain an AP fragment represented in KinFragLib (red) and CustomKinFragLib (green) are colored. The point size is scaled according to the number of ligands from this kinase that are represented in the fragmentation library. Note that if the number of ligands per kinase in KinFragLib and CustomKinFragLib is the same, the point will only be displayed in green.

As shown, all major kinase families are represented, and the majority of kinases (87%) still contain ligand-derived fragments in one or more subpockets of our reduced fragmentation library. Out of 326 kinases recorded in KLIFS, the number of kinases covered by AP subpocket fragments is reduced from 203 in KinFragLib to 127 in CustomKinFragLib. Nevertheless, a substantial portion of the original coverage is retained (see Table 4). Our reduced fragmentation library continues to provide broad coverage of ligands across kinase families and subpockets, except the back pockets (B1 and B2). These subpockets contain relatively few fragments overall, so limited coverage is expected here.

**Table 4:** Number of Kinases Covered in Pre-Filtered KinFragLib Compared to CustomKinFragLib^a^

Subpocket	Prefiltered KinFragLib	CustomKinFragLib
AP	203	127
FP	183	113
SE	157	104
GA	154	98
B1	35	13
B2	30	17
Total	203	177

a A kinase is considered to be represented, if at least one fragment originating from a ligand binding to this kinase is included in the respective fragment library.

To further assess the chemical diversity of the represented kinase ligands, we visualized the chemical space of all KLIFS ligands with at least one fragment included in KinFragLib or CustomKinFragLib (Figure 15). Despite the strong reduction in fragment count, CustomKinFragLib retains fragments from a large and diverse set of original kinase ligands.

**Figure 15:**
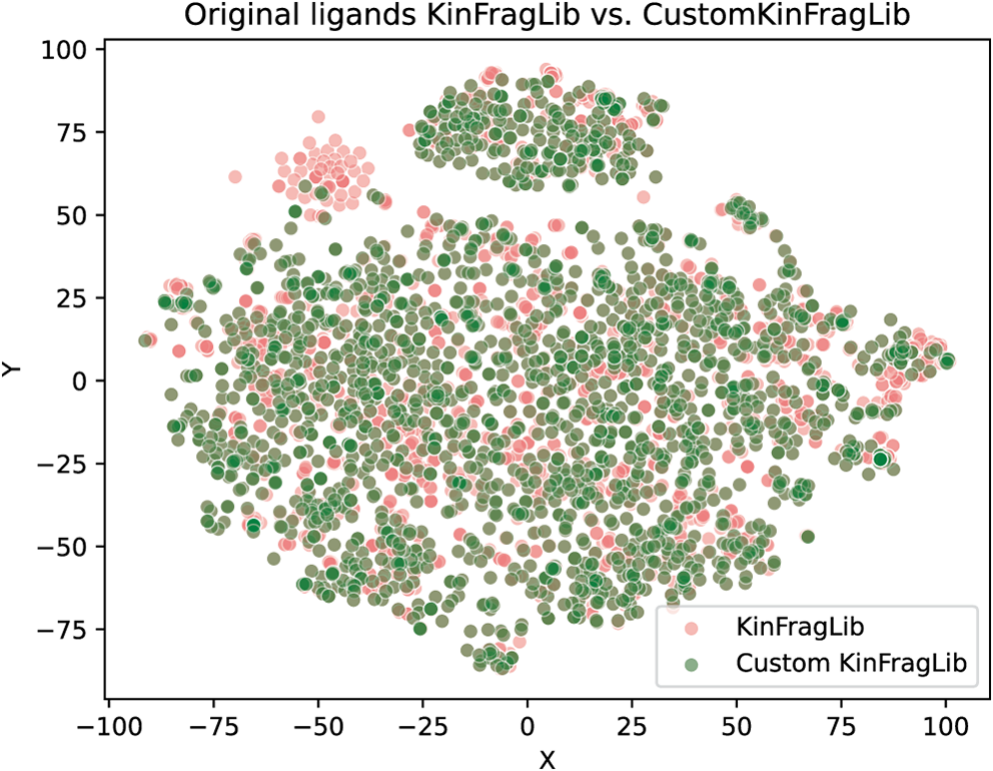
t-SNE plot of the chemical space of original kinase ligands from KLIFS^4^ represented in KinFragLib. A ligand is considered to be represented if at least one fragment is included in KinFragLib (red) and CustomKinFragLib (green), respectively.

### Analysis of Enumerated Molecules

To assess whether fragment-level filtering translates into improved molecule-level properties, we compared two recombination sets: molecules generated from CustomKinFragLib fragments and molecules built from fragments rejected during the filtering pipeline. We investigate drug-likeness and synthesizability across both sets (see Figure 16).

**Figure 16:**
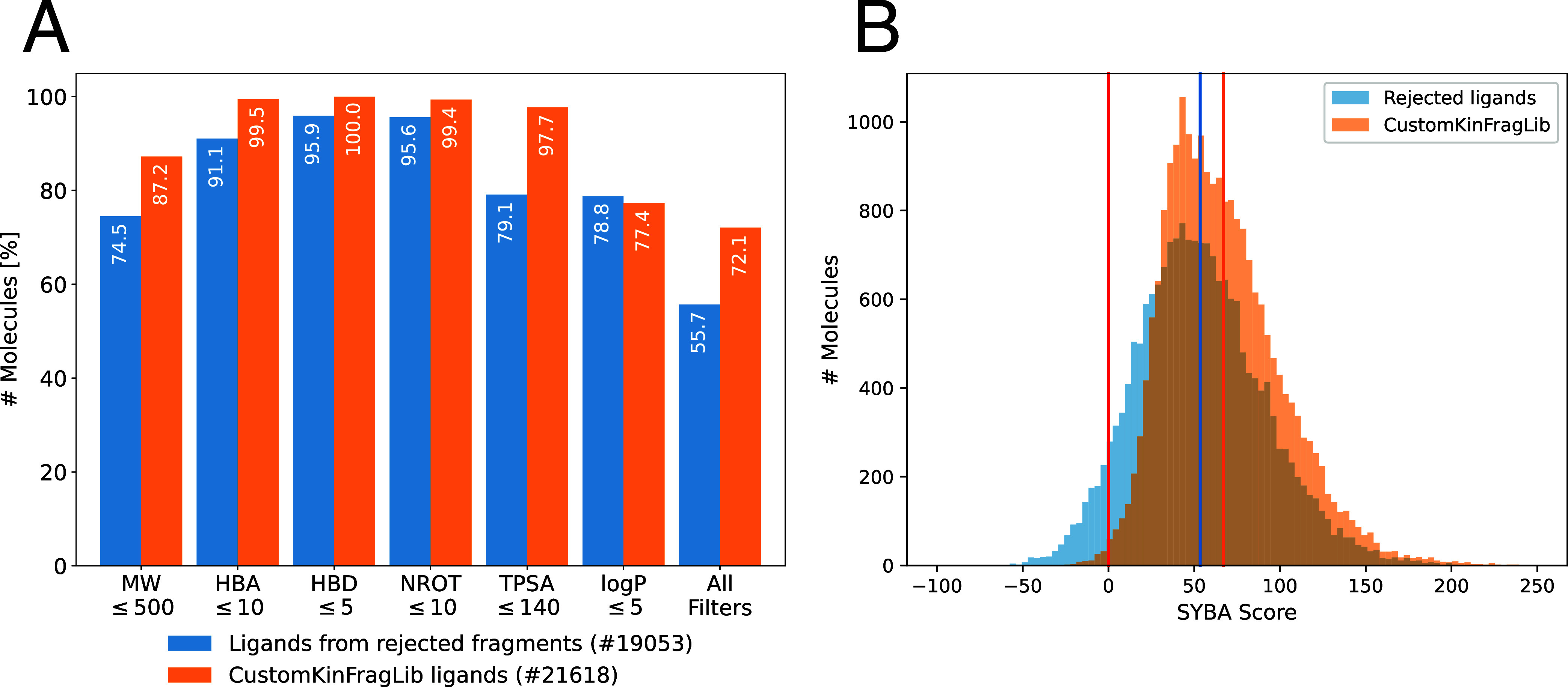
(A) Fraction of molecules passing each filter for CustomKinFragLib ligands and ligands built from fragments rejected in CustomKinFragLib. (B) SYBA score distribution of CustomKinFragLib ligands (orange) and rejected ligands (blue). The mean of both distributions is indicated in the respective color. The red line indicates the threshold used in CustomKinFragLib.

Across nearly all criteria (except log *P*), a larger fraction of CustomKinFragLib molecules satisfied the filters compared to those derived from rejected fragments. While 72% CustomKinFragLib molecules fulfill all drug-likeness filters, only 56% of molecules from rejected fragments fulfill all filters (see Figure 16A). We also calculated the QED score for all molecules and compared the distributions, and we observed a favorable shift in CustomKinFragLib ligands toward more drug-like molecules (see Supporting Figure S6A).

Synthesizability was assessed using the SYBA score and similarity to the Enamine REAL Space. CustomKinFragLib molecules showed markedly higher SYBA scores, with most values above the CustomKinFragLib threshold, indicating improved predicted synthetic accessibility. Additionally, a much larger fraction of molecules from the rejected fragments (≈6%) are below the threshold (compared to ≈0.5% for CustomKinFragLib). Comparison with the molecules in the Enamine REAL Space using SpaceLight further supports this trend: the distribution of closest-match Tanimoto similarity was consistently higher for CustomKinFragLib ligands (see Supporting Figure S6B), aligning with the fragment-level filtering against Enamine building blocks. Overall, all evaluated molecular properties improved after filtering, suggesting that filtering on a fragment level improves the molecular properties of recombined ligands.

### Exemplary Demonstration of Practical Usage of CustomKinfragLib

CustomKinFragLib has already been successfully used in a PKA kinase ligand design study by Buchthal et al.,^35^ where a fragment-based, subpocket-guided docking pipeline was developed to identify novel kinase ligands. In this study, CustomKinFragLib has served as the starting fragmentation library for iterative fragment growing. Because the workflow in that study employed a novel greedy search strategy enabling exploration of a larger chemical space, and because the goal was to synthesize selected molecules in a collaborating laboratory rather than purchasing them from Enamine, the filtering parameters were adjusted accordingly, highlighting the flexibility of our pipeline. Using the CustomKinFragLib-derived fragment set, the authors generated and evaluated approximately 59,000 recombined ligands, from which several candidates were selected for synthesis. Fifteen compounds were successfully synthesized, out of which seven showed promising inhibitory activity against PKA. Moreover, crystallographic analysis of the most potent compound confirmed that the experimentally observed binding mode closely overlapped with the predicted docking pose, demonstrating the practical relevance of the recombined structures. This prospective application illustrates how CustomKinFragLib can provide a chemically meaningful and synthetically tractable starting point for fragment-based kinase inhibitor design. For full methodological details and experimental results, we refer readers to Buchthal et al.^35^

## Conclusion

Protein kinases play a crucial role in diseases such as cancer and autoimmune disorders, making them vital drug targets. To meet the challenge of designing novel kinase inhibitors, FBDD has shown great promise. The kinase-specific fragment library KinFragLib is a data-driven FBDD approach providing a powerful subpocket-specific framework for creating potentially feasible kinase inhibitors through subpocket-guided enumeration and combination of fragments. However, traversing the entire recombination space of KinFragLib is computationally infeasible, necessitating a reduced version.

We therefore introduced CustomKinFragLib, a kinase-specific fragment library, which focuses on a subset of promising fragments, creating a more computationally feasible fragment space. We achieve this by filtering the fragment space based on unwanted substructures, favorable molecular properties, and synthetic feasibility.

Synthesizability scores are commonly used to estimate how easily novel molecules can be synthesized, thanks to their fast and straightforward application. While useful, especially ML-based scores can have limited generalizability due to data sparsity and bias.^36^ Since these scores do not suggest specific synthesis routes, they are best used alongside synthesis planning tools such as ASKCOS. Additionally, the commercial availability of the fragments is crucial to speed up the drug discovery process. We integrated a substructure search for the Enamine building blocks, but ideally, querying other major commercial databases (MolPort,^37^ eMolecules,^38^ Chemspace,^39^ to name a few) would further improve our synthetic feasibility filtering. The implemented filter can also be adapted to prioritize readily accessible compounds by restricting the substructure search to building blocks explicitly listed as in stock.

By applying our filtering pipeline, we reduced the fragment space from 9131 fragments to 837 fragments while retaining a high diversity. CustomKinFragLib is available as a user-friendly Python package. The different filters can easily be adapted or excluded by the user to generate a more strict or wider fragment library, depending on the application and personal preference. The resulting focused fragment library offers a more effective starting point for the design of novel kinase inhibitors, combining meaningful chemical diversity with improved synthetic accessibility.

## Supplementary Material



## Data Availability

The fragmentation library and the results described are openly available on GitHub at https://github.com/volkamerlab/KinFragLib (also available on Zenodo: 10.5281/zenodo.17659926). Further data underlying this study are openly available on Zenodo at 10.5281/zenodo.18386001.

## Notes

Data and Methods: Retrosynthesis parameters used for the ASKCOS query (Table S1); number of fragments and molecules in enumeration sets (Table S2). Results: Rule of Three (Ro3) filters for example molecules (Table S1); distribution of SYBA scores for fragments across the different subpockets (Figure S1); distribution of QED scores for drug-like fragments and for KinFragLib fragments (Figures S2 and S3); chemical space analysis for each filter (Figure S4); kinase coverage of our fragmentation libraries across the kinome tree (Figure S5); molecular property distributions for enumeration sets (Figure S6) (PDF)
