# Supplementary material for: CustomKinFragLib: Filtering the Kinase-Focused Fragmentation Library
Source: ACS Omega. 2026 Mar 6;11(10):16369–80. doi: 10.1021/acsomega.5c12231 (PMC13000600; doi:10.1021/acsomega.5c12231)
Supplement: Supplementary file 1 [file ao5c12231_si_001.pdf]

Supporting Information: CustomKinFragLib: Filtering the Kinase-Focused Fragmentation Library

Paula Linh Kramer,[†] Katharina Buchthal,[†] Dominique Sydow,[‡] Katharina Sonja Leo,[‡] and Andrea Volkamer[*,†,‡]

†*Data Driven Drug Design, Center for Bioinformatics, Saarland University, Campus, 66123 Saarbrücken, Germany*

‡*In Silico Toxicology and Structural Bioinformatics, Institute of Physiology, Charité - Universitätsmedizin Berlin, Charitéplatz 1, 10117 Berlin, Germany*

* E-mail: volkamer@cs.uni-saarland.de

Data and Methods

Table S1: Retrosynthesis parameters used for the ASKCOS[1] query in the jupyter notebook.

ASKCOS parameter	Value	Description
max_depth	1	Number of retrosynthetic steps
max_branches	25	Number of branches looked at to find the best
expansion_time	20	Seconds the query is running
max_ppg	100	Maximum price per gram for buyable compounds
template_count	100	Maximum number of templates looked at
max_cum_prob	0.995	Minimum probability until no more templates are used
chemical_property_logic	“none"	Only the price of the molecule is relevant, not the number of heavy atoms
chemical_popularity_logic	“none"	Do not use popular chemicals as reasonable stopping points
return_first	“true"	Return results that are found not only the best

Table S2: The number of fragments sampled per subpocket for both enumeration sets, followed by the number of enumerated molecules using the sampled subpocket fragments. In the final step, full molecules with different number of fragments are sampled, to create a chemical space similar to known kinase ligands.

Subpocket	CustomKinFragLib Set	Rejected Set
AP	80	80
FP	80	80
SE	80	80
GA	80	80
B1	18	29
B2	26	33
Enumerated molecules	786,212	774,070
Sampled molecules	21,618	19,053

Results

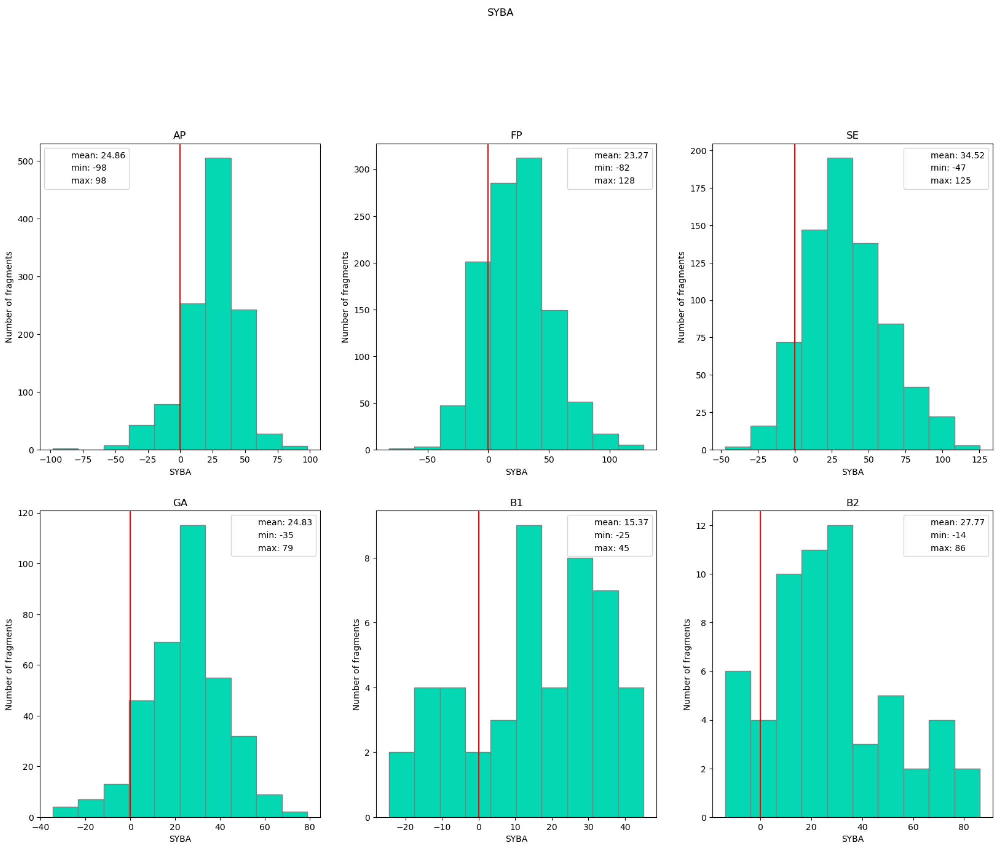

Figure S1: Distribution of SYBA[2] scores for each subpocket. The red line indicates the default filtering cutoff.

Table S3: Example molecules from Figure 8 in the main manuscript with all Rule of Three they pass (T) and fail (F).

	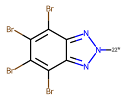	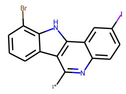	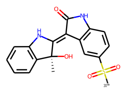	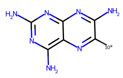	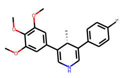	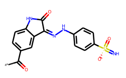
MW< 300	F	F	F	T	F	F
LogP ≤ 3	F	F	T	T	F	T
HBD ≤ 3	T	T	F	T	T	T
HBA ≤ 3	T	T	F	F	F	F
ROTB ≤ 3	T	T	T	T	F	F
PSA ≤ 60	T	T	F	F	T	F

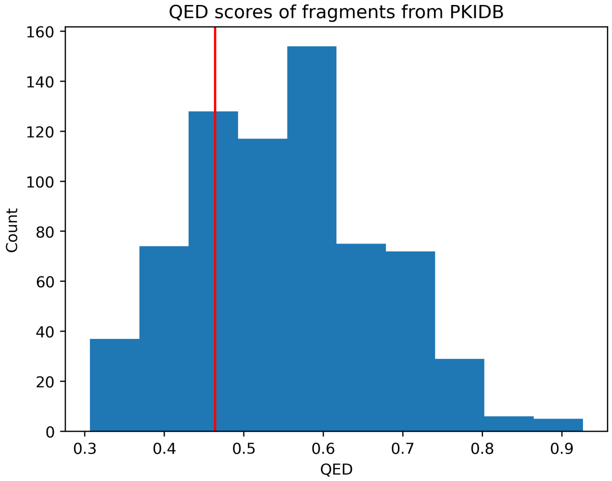

Figure S2: QED scores of drug-like fragments to establish default threshold. We take FDA-approved kinase inhibitors and inhibitors in clinical trials from PKIDB[3] to extract the KinFragLib fragments. The lower 25% quantile of the QED scores is taken as a default threshold (red line).

QED

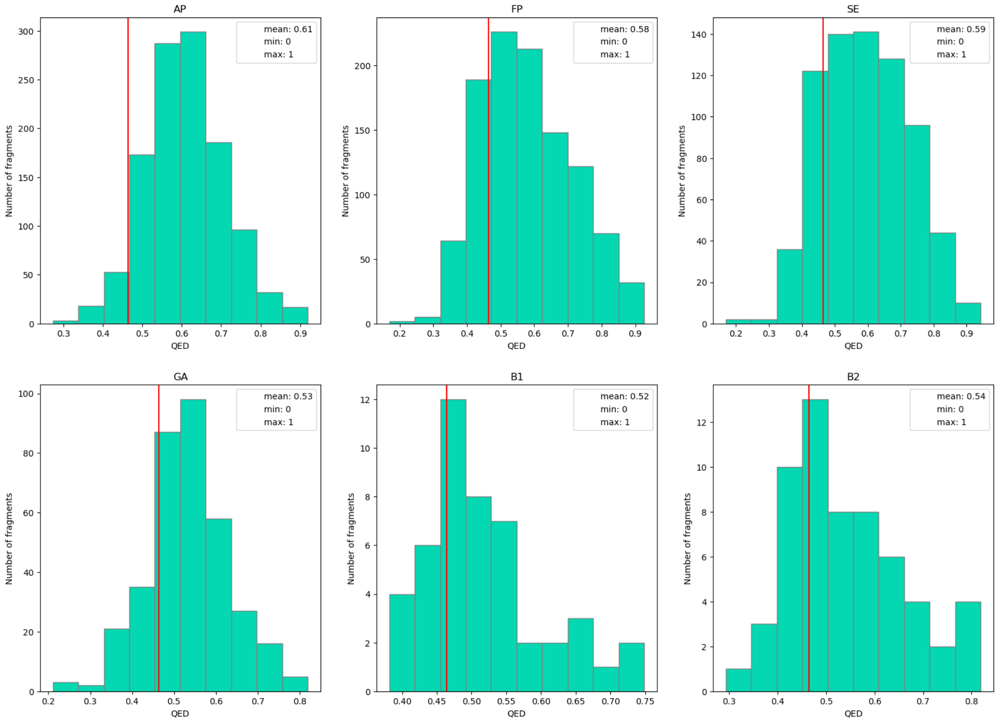

Figure S3: Distribution of QED[4] scores for each subpocket. The red line indicates the default filtering cutoff (0.464).

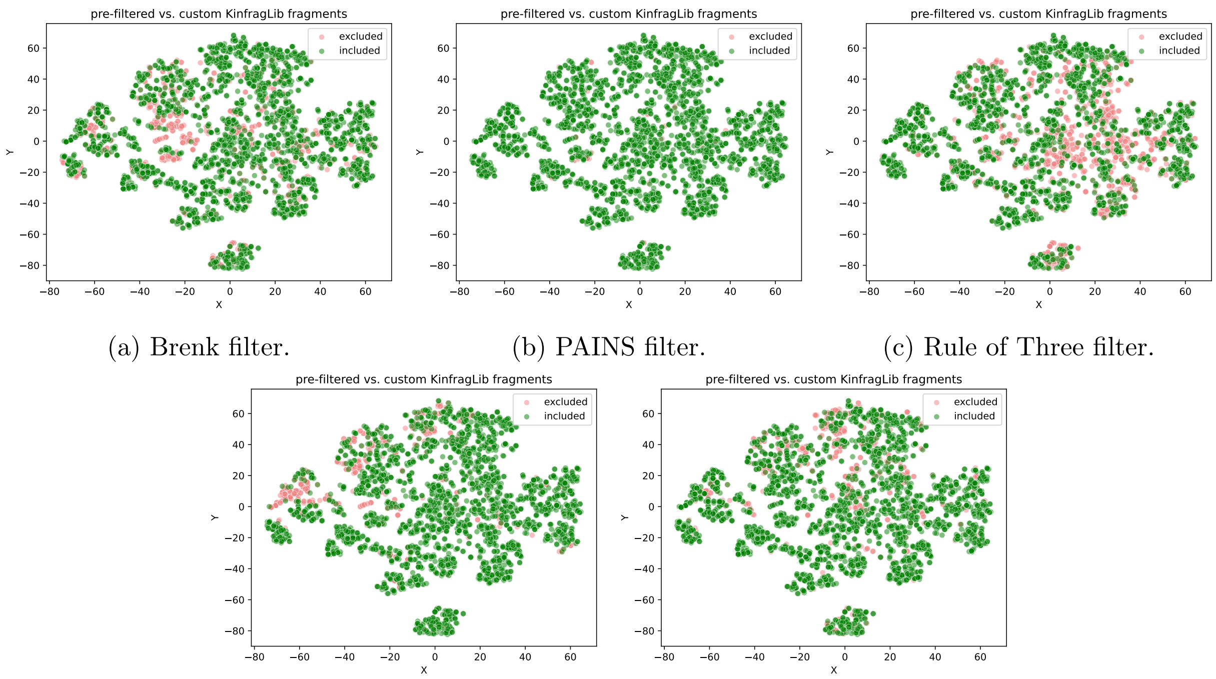

(a) Brenk filter.

(b) PAINS filter.

(c) Rule of Three filter.

(d) QED filter.

(e) SYBA filter.

Figure S4: t-SNE of pre-filtered fragments for all filtering steps separately. Fragments in green fulfill the filter criteria stated in the caption.

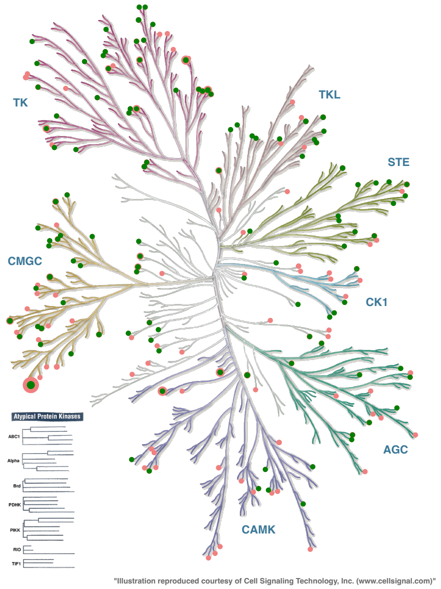

(a) FP subpocket.

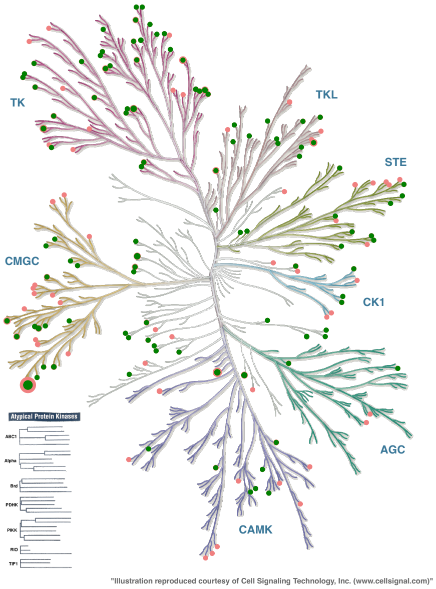

(b) SE subpocket.

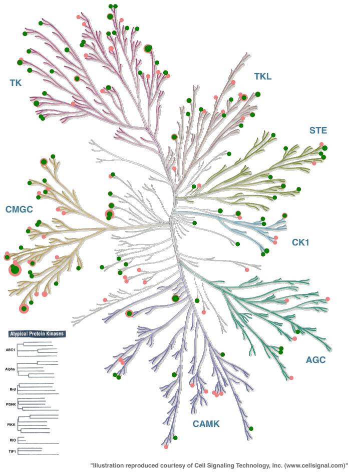

(c) GA subpocket.

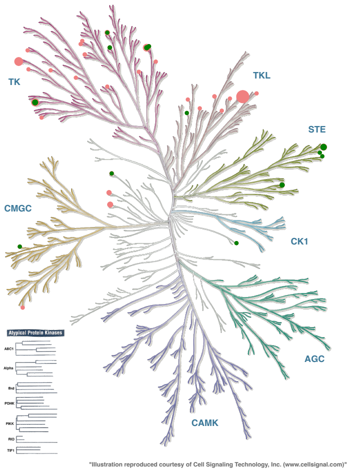

(d) B1 subpocket.

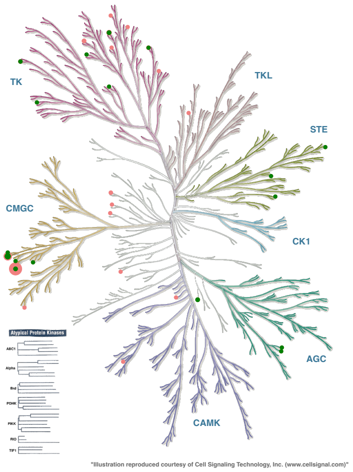

(e) B2 subpocket.

Figure S5: Kinome tree generated using KinMap.[5] Ligands binding to the kinases that contain a fragment represented in KinFragLib (red) and CustomKinFragLib (green) are colored. The point size is scaled per subpocket according to the number of ligands from this kinase that are represented in the fragmentation library. Note that if the number of ligands per kinase in KinFragLib and CustomKinFragLib is the same, the point will only be displayed in green.

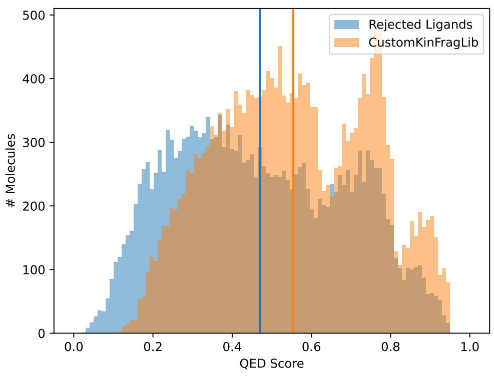

(a)

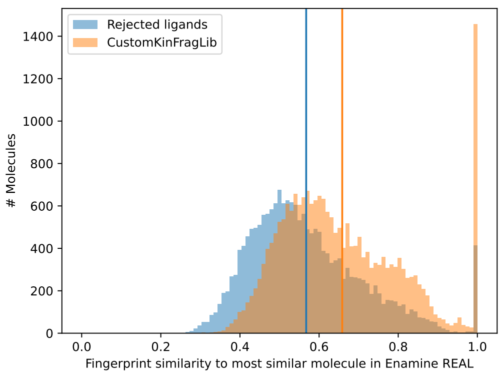

(b)

Figure S6: (a) QED scores for enumerated molecules from CustomKinFragLib ligands (orange) and ligands recombined from rejected fragments (blue). The means are added in the respective colors. (b) Fingerprint similarity of each CustomKinFragLib ligand (orange) to the most similar molecule in the Enamine REAL Space, and the similarity of rejected ligands (blue) to the most similar Enamine molecule. The mean is displayed in the respective colors.

References

(1) ASKCOS. https://gitlab.com/mlpds_mit/askcosv2/askcos2_core, Accessed: 2024-01-22.

(2) Voršilák, M.; Kolář, M.; Čmelo, I.; Svozil, D. SYBA: Bayesian estimation of synthetic accessibility of organic compounds. *Journal of cheminformatics* **2020**, *12*, 1–13.

(3) Carles, F.; Bourg, S.; Meyer, C.; Bonnet, P. PKIDB: A curated, annotated and updated database of protein kinase inhibitors in clinical trials. *Molecules* **2018**, *23*, 908.

(4) Bickerton, G. R.; Paolini, G. V.; Besnard, J.; Muresan, S.; Hopkins, A. L. Quantifying the chemical beauty of drugs. *Nature chemistry* **2012**, *4*, 90–98.

(5) Eid, S.; Turk, S.; Volkamer, A.; Rippmann, F.; Fulle, S. KinMap: a web-based tool for interactive navigation through human kinome data. *BMC bioinformatics* **2017**, *18*, 16.
